# Zeta Sperm Selection Improves Pregnancy Rate and Alters Sex Ratio in Male Factor Infertility Patients: A Double-Blind, Randomized Clinical Trial 

**DOI:** 10.22074/ijfs.2016.4917

**Published:** 2016-06-01

**Authors:** Mohammad Hossein Nasr Esfahani, Mohammad Reza Deemeh, Marziyeh Tavalaee, Mohammad Hadi Sekhavati, Hamid Gourabi

**Affiliations:** 1 Department of Reproductive Biotechnology, Reproductive Biomedicine Research Center, Royan Institute for Biotechnology, ACECR, Isfahan, Iran; 2 Isfahan Fertility and Infertility Center, Isfahan, Iran; 3 Department of Animal Science, Ferdowsi University of Mashhad, Mashhad, Iran; 4 Embryonic and Stem Cell Biotechnology Research Group, Institute of Biotechnology, Ferdowsi University of Mashhad, Mashhad, Iran; 5 Department of Genetics, Reproductive Biomedicine Research Center, Royan Institute for Reproductive Biomedicine, ACECR, Tehran, Iran

**Keywords:** Zeta Potential, Density Gradient Centrifugation, Sex Ratio, Embryo Quality, Pregnancy

## Abstract

**Background:**

Selection of sperm for intra-cytoplasmic sperm injection (ICSI) is usually considered as the ultimate technique to alleviate male-factor infertility. In routine ICSI, selection is based on morphology and viability which does not necessarily preclude the chance injection of DNA-damaged or apoptotic sperm into the oocyte. Sperm with high negative surface electrical charge, named “Zeta potential”, are mature and more likely to have intact chromatin. In addition, X-bearing spermatozoa carry more negative charge. Therefore, we aimed to compare the clinical outcomes of Zeta procedure with routine sperm selection in infertile men candidate for ICSI.

**Materials and Methods:**

From a total of 203 ICSI cycles studied, 101 cycles were allocated to density gradient centrifugation (DGC)/Zeta group and the remaining 102 were included in the DGC group in this prospective study. Clinical outcomes were com- pared between the two groups. The ratios of Xand Y bearing sperm were assessed by fluorescence *in situ* hybridization (FISH) and quantitative polymerase chain reaction (qPCR) methods in 17 independent semen samples.

**Results:**

In the present double-blind randomized clinical trial, a significant increase in top quality embryos and pregnancy rate were observed in DGC/Zeta group compared to DGC group. Moreover, sex ratio (XY/XX) at birth significantly was lower in the DGC/Zeta group compared to DGC group despite similar ratio of X/Y bearings sper- matozoa following Zeta selection.

**Conclusion:**

Zeta method not only improves the percentage of top embryo quality and pregnancy outcome but also alters the sex ratio compared to the conventional DGC method, despite no significant change in the ratio of Xand Ybearing sperm population (Registration number: IRCT201108047223N1).

## Introduction

Intra-cytoplasmic sperm injection (ICSI) is usually considered as the ultimate technique to alleviate male-factor infertility when other assisted reproductive technologies (ART) fail to help a couple conceive. During ICSI, a single sperm is directly deposited into the cytoplasm of a mature oocyte, thereby bypassing all natural selection barriers to fertilization (1). 

Accordingly, studies demonstrate that selection of sperm based on viability and morphology does not necessarily preclude the chance of oocyte injection with a DNA-damaged or apoptotic sperm when there are no other criteria for selection of sperm in conventional ICSI procedure (2,3). 

To address this obstacle, a series of advanced strategies for non-invasive selection of intact sperm based on cellular and molecular principles have been implemented ( for more detail see review by Nasr-Esfahani et al. (4), and Simon L et al. (5). In this regard, Chan et al. (6) and our group (7) proposed that sperm population selected based on the membrane Zeta potential represent lower degrees of DNA fragmentation. Zeta potential is a negative electro-kinetic potential of around -16 to -20 mV which is acquired by sperm-membrane during spermatogenesis and epididymal maturation as a result of sperm surface coating with sialic acids (8). 

Our recent study has provided preliminary data on the capacity of Zeta potential to improve the ICSI outcomes on small population (9). Therefore, we aimed to compare clinical outcomes of ICSI using sperm selected by using of Zeta potential or routine density gradient centrifugation (DGC) methods. Moreover, considering differential Zeta-potential of Xand Ybearing sperm (10), we designed to understand whether Zeta method of sperm selection has any bearing influence on the sex ratio of developed pregnancies developed by ICSI. In the present double-blind randomized clinical trial, we showed that Zeta procedure not only improves the pregnancy outcome but also alters the sex ratio of developed pregnancies, despite no significant change in the ratio of Xand Ybearing sperm. 

## Materials and Methods

### Patients

This prospective study was approved by the Research Ethics Committee involving human subjects at Royan Institute and Isfahan Fertility and Infertility Center. A total of independent 228 ICSI cycles were included in a parallel double-blind randomized clinical trial spanning the period between September 2010 and March 2014. The power of sample size was calculated to be around 200 based on a previous study (9). Furthermore, we assessed the ratio of Xand Ybearing sperm by fluorescence *in situ* hybridization (FISH) and quantitative polymerase chain reaction (qPCR) methods in 17 independent samples of all 228 semen samples subjected to DGC/Zeta and DGC procedures. 

### Inclusion criteria

A trained nurse was asked to assess the last ultrasound and semen analysis of ICSI candidates on the day of human chorionic gonadotropin (hCG) injection. Accordingly, women below 40 years who had adequate number of follicle in their last ultrasound scan (at least 4 dominate follicle greater 16 mm) and at least one semen parameter (volume, total motility, progressive motility, concentration and morphology) of their partner was below normal threshold based on World Health Organization (WHO 2010) (11). The verified couples were randomly allocated using block designed between the control (DGC) or treatment (DGC/Zeta) trial groups by one of the staff who was unaware of the experimental study. On the day of ICSI, semen samples from men were assessed according to WHO (2010) (11) and only this data for semen samples are provided in this study. 

### Exclusion criteria

Women with poor quality oocyte (abnormal zona pellucida, large perivitelline space, refractile bodies, increased cytoplasmic granularity, smooth endoplasmic reticulum clusters, and abnormal, fragmented, or degenerated polar bodies) and endometrial thickness greater than 7 mm (type C) were excluded from this study. 

### Semen processing by density gradient centrifugation

All procedures were conducted under sterile conditions. Semen processing was carried out using Ham’s F-10 supplemented with 10% human serum albumin (HAS, Octalbin, Switzerland). Liquefied semen samples were placed on PureSperm column (80% lower, 40% upper) and centrifuged at 300 g for 20 minutes. Sperm pellets were suspended in Ham’s-F10 plus albumin and washed twice in the same medium. The pellet was finally resuspended in 1 ml of the Ham’s-F10 plus albumin for ICSI. 

### Sperm selection based on combined density gradient and Zeta

The Zeta method was carried out according to modified protocol of Chan et al. (6). For DGC/ Zeta, Ham’s-F10 was used without serum supplementation, unless otherwise stated. Immediately after DGC, sperm pellets were washed with Ham’s-F10, re-suspended and diluted in 4 ml Ham’s -F10 in 5 ml Falcon plastic tubes. The prepared sperm suspension was subsequently exposed to the positive charge which was induced by placing the tube inside a latex glove up to the cap. For induction of the charge, the glove was rotated or twisted two or three turns around the tube which was grasped by its cap. Finally, the tube was rapidly removed from the glove and kept at room temperature for 1 minute to allow adherence of the "intact" sperm to the charged tube wall. The medium then was dispensed from the tube to eliminate any non-adhering sperm and the tube wall was washed with 4 ml Ham’sF10 plus albumin to neutralize the charge on the tube wall and to detach adhering sperm. The tube was centrifuged and the pellet was re-suspended in 1 ml of Ham’s-F10 plus albumin to be used for ICSI. The entire centrifugation step was carried out at 300 g for 5 minutes. For verification of Zeta procedure, an electrostatic voltmeter (Alpha lab, Salt Lake City, USA) was used (7). To minimize variation, a trained individual carried out all procedures and the tubes were labeled by codes. In addition, the embryologist who performed the ICSI procedure was unaware of the individual allocation to the groups (DGC or DGC/Zeta) or the type of sperm preparation implemented. 

### Intra-cytoplasmic sperm injection

A single standard stimulation and ovulation induction protocol, and ICSI procedure were performed for all the cases (9). 

Fertilization rate was calculated from the ratio of fertilized oocytes (2PN) by the total number of injected metaphase II oocytes, multiplied by 100. Embryo quality was assessed by a certain staff who was not involved and aware of trail on day 3 post-oocyte retrieval and a top quality embryo was defined as an embryo between 6-8 cells with equal blastomere size and less than 25% fragmentation (12,13). Percentage of top quality embryos was assessed by dividing number of top quality embryo by the total number of embryos, multiplied by 100. Chemical pregnancy was defined when β-hCG level was higher than 10 IU and clinical pregnancy rate was defined by ultrasonography findings showing at least one embryo with a fetal heart beat, 5 weeks after transfer. Implantation rate was defined by the number of observed gestational sacs per number of transferred embryos. 

### Assessing Xand Ybearing sperm ratio

Ratio of Xand Ybearing sperm was determined by FISH technique according to Aleahmad et al. (14). Quantitative PCR was also conducted according to Ainsworth et al. (15) 2011 for determining the ratio of X and Y bearing sperm. 

### Quantitative polymerase chain reaction method

Genomic DNA was extracted using the DNeasy® Blood & Tissue Kit (Qiagen^TM^, Germany), according to the manufacturer’s instructions with some modifications. In brief, semen and blood samples were centrifuged at 3000 g for 3 minutes. The sperm pellet was re-suspended in 200 μl phosphatebuffered saline (PBS) and the samples were treated with proteinase K (40 mAU/mg protein, supported by DNeasy® Blood & Tissue Kit) and incubated at 56°C for 30 minutes. Genomic DNA was harvested by Mini spin column and stored at -20°C. Sex determining region Y (*SRY*) and *Amelogenin* genes were candidate as Y and X chromosome determinations, respectively. SRY gene located on p11.3 region of the Y chromosome encodes a transcription factor that belongs to the high mobility group (HMG) box that has a DNA binding domain and was used as a dominant gene in mammalian male sex determination (16). *Amelogenin* gene is located on the X and Y chromosomes at X p22.1X p22.3 and Y p11.2. This gene could be used as a sequence for mammalian female sex determination because it has a 177-bp fragment which inserted just in X-sequence (15). To amplify Y and X specific chromosome fragments by PCR, two pairs of primers were designed (Table 1). 

**Table 1:** The list of primers used in this study


Primer	Sequence (5'-3')	Size	Gene	Accession no.

SRY	F: CGTCGGAAGGCGAAGATGC	167-bp	SRY	NW_001842360.1
	R: TTGATGGGCGGTAAGTGGC			
Amel	F: GTGTCTCTTGCTTGCCTCTGC	107-bp	Amelogenin	NW_001842422.1
	R: GGAGAACCTCAAACCCGACG			


**Fig.1:**
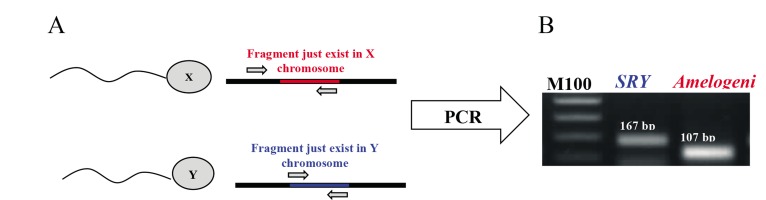
PCR amplicons of the *Amelogenin* and *Sex* determining region Y (SRY) gene from sperm genomic DNA. A. Schematic illustration of annealing of primers for specific amplification of X and Y chromosome in PCR reaction and B. A 167-bp and 107-bp fragment was amplified in PCR reaction from *SRY* and *Amelogenin* gene as indicators for sex determination. PCR; Polymerase chain reaction.

SRY-forward/reverse primers were designed to amplify a 167-bp fragment from SRY gene. For amplification of an X chromosome specific fragment, Amel-reverse primer was designed to anneal to inserted 177-bp fragment in X-sequence. Amel-forward/reverse primers amplified a specific 107-bp fragment for X chromosome (Fig .1). Sex ratio was quantified by quantitative real-time PCR (RT-qPCR) using the Rotorgene 2000 Real Time Cycler (Corbett Research, Sydney, Australia). For each sample, RT-qPCR was performed in triplicate. PCR was conducted by adding 1 µL genomic DNA to the 20 µL of PCR mixture that contained 1 ×SYBR® Premix Ex Taq^TM^(Takara Bio Inc., Otsu, Japan), 0.4 µM of each specific primer, and DNase-free water. The PCR protocol included an initial step of 94°C (4 minutes), followed by 40 cycles of 94°C (30 seconds), 60°C (30 seconds), and 72°C (30 seconds). Primer efficiency was evaluated by making a 5-fold serial dilution of each samples reaction for each primer pairs and was calculated by 10-1/slope equation. The sex ratio in each reaction was calculated by the ratio of threshold cycle (CT) of X to Y (X/Y). 

### Fluorescence in situ hybridization technique

Mixture of X (DXZ1) red and Y (DYZ3) green (Abbott) labeled probes were prepared for detection of X and Y chromosomes in sperm nuclei. Under a cover slip, ten microliter of probe mixture was added and sealed with rubber cement. For hybridization of the DNA probes, spermatozoa and probe DNA were concomitantly denatured for 5 minutes at 75ºC. Then, slides were incubated in a moist chamber at 37ºC for 4 hours. After washing with 0.4× SSC/0.3% NP40 at 73ºC for 2 minutes and then in 2× SSC/0.1% NP40 at room temperature for 1 minute, slides were counterstained with 10 ml of 4(6-diamidino-2-phenylindole) (DAPI) mixed with antifade (Cytocell Technologies Ltd, UK) using a fluorescence microscope (Nikon E800, Japan) equipped with a triple-band 476 pass filter for DAPI/ spectrum green/spectrum orange. At least 1000 spermatozoa with intact nuclei were counted and green or orange fluorescent spot were considered as X and Y chromosomes in sperm, respectively (14). 

### Statistical analysis

For statistical analysis, the Chi-square, Student’s t test, one-way analysis of variance (ANOVA) and logistic regression model were carried out using the Statistical Package for the Social Sciences software (SPSS 18, Chicago, IL, USA). All data were presented as means ± SEM, and differences were considered significant at P<0.05.

## Results

### Patient cohorts

251 couples were informed about the trial and 228 accepted to participate in the trail. Due to technical limitation, two cases were included per day (one for DGC and one for DGC/Zeta) and therefore, 20 cases which had the inclusion criteria were excluded from the study. Of the 208 remaining cases, 5 cases (3 from DGC/Zeta and 2 from control group) were excluded from the study based on the exclusion criteria. Of the 203 ICSI cycles included in this study, 101 cases were designated to the DGC/Zeta group while 102 were allocated to DGC group. 

### Confounding factors

Table 2 compares possible confounding factors between DGC/Zeta and DGC groups. As shown, no significant difference in term of semen parameters, number of oocyte retrieved (Table 3), female and male ages were observed between the two groups. 

**Table 2:** Comparison of possible confounding factors between DGC/Zeta and DGC groups


	DGC/Zeta group Mean (SE) n=102	DGC group Mean (SE) n=101	P value

Male age (Y)	35.76 ± 5.91	36.79 ± 6.18	0.22
Sperm concentration (10^6^/ml)	44.27 ± 3.42	42.14 ± 3.43	0.41
Total sperm motility(%)	38.84 ± 1.20	39.09 ± 1.45	0.89
Progressive motility (%)	16.86 ± 1.05	16.21 ± 1.24	0.68
Sperm normal morphology (%)	3.78 ± 0.18	4.13 ± 0.15	0.12
Female age (Y)	30.73 ± 0.48	31.34 ± 0.53	0.26


DGC; Density gradient centrifugation.

### Intra-cytoplasmic sperm injection outcomes

Table 3 shows ICSI outcome between the two groups. No difference in fertilization rates was observed between the DGC/Zeta and DGC groups (77.89 ± 1.87 vs. 76.91 ± 2.08%, respectively). Even though, the respective percentages of top quality embryos (45.83 ± 3.11 vs. 35.38 ± 4.64%), chemical pregnancy (43.13 vs. 23.7%), clinical pregnancy (39.2 vs. 21.8%) and abortion (7.5 vs. 18.2%) were significantly improved in DGC/Zeta group when compared with DGC group. The implantation rate was similar between the two groups (21.01 vs. 12.75% in DGC/Zeta and DGC group, respectively). The mean numbers of embryos transferred were 2.51 ± 0.08 vs. 2.48 ± 0.09 in DGC/Zeta and DGC group, respectively without any significant difference. 

**Table 3:** Comparison of ICSI outcome between DGC/Zeta and DGC groups


	DGC/Zeta group n=102	DGC group n=101	P value

Number of oocyte retrieved	8.65 ± 0.40	8.06 ± 0.35	0.17
Fertilization rate (%)	77.89 ± 1.87	76.91 ± 2.08	0.72
Top quality embryo (%)	45.83 ± 3.11	35.38 ± 4.64	0.04*
Mean of transferred embryos	2.51 ± 0.08	2.48 ± 0.09	0.78
Mean of vitrified embryos	2.06 ± 0.26	1.78 ± 0.25	0.45
Chemical pregnancy rate (%)	44/102 (43.13%)	24/101 (23.7%)	0.004*
Clinical pregnancy rate (%)	40/102 (39.2%)	22/101n(21.8%)	0.009*
Abortion rate (%)	3/40 (7.5%)	4/22 (18.2%)	0.03*
Stillbirth rate (%)	0(0%)	2(18.2%)	0.00*
%Implantation rate (%)	54/257 (21.01%)	32/251 (12.75%)	0.13


Independent studentʼs t test and Chi-square carried out for statically analyzing. ICSI; Intra cytoplasmic sperm injection, DGC; Density gradient centrifugation, and *; Indicates significant dif- ference (P<0.05).

### Confounding factors of intra-cytoplasmic sperm injection outcomes

To compare the clinical pregnancy rate between the two groups and evaluate the possible con- founding factors on ICSI outcomes, we applied binary regression model (Table 4). Results showed the odds ratio of clinical pregnancy between DGC/ Zeta versus DGC group was 2.304 with P=0.01. Therefore, the chance of clinical pregnancy rate in DGC/Zeta group was 2.3 fold higher than DGC group. These data revealed that confounding fac- tors which had significant influence on the ICSI outcome were male smoking, female age, total oocyte retrieved and injected, ovarian factor and polycystic ovarian syndrome.

**Table 4:** Multiple regression analysis for DGC vs. DGC/Zeta


Parameters	P value	Odds ratio	95% CI for EXP(B)	
			Lower	Upper

Male smoking	0.042^*^	0.383	0.152	0.965
Female age	0.012^*^	0.903	0.834	0.978
Total oocyte retrieved	0.019^*^	0.795	0.656	0.963
Injected oocyte	0.020^*^	1.290	1.040	1.601
Tubal factor	0.608	1.238	0.547	2.805
Endometriosis	0.248	2.075	0.601	7.165
Uterine factor	0.798	1.143	0.410	3.185
Polycystic ovarian Syn.	0.005 *	5.618	1.699	18.577
Ovarian factor	0.049^*^	0.352	0.124	1.002
Duration of infertility	0.293	0.743	0.427	1.292
No. Previous ART	0.994	1.000	0.914	1.093
Clinical pregnancy (DGC/Zeta vs. DGC)	0.018 *	2.304	1.154	4.601


Binary logistic regression carried out for statically analyzing. ^*^; Indicates statistical significance (P<0.05), CI; Confidence interval, DGC; Density gradient centrifugation, and ART; Assisted reproduction technique.

### Sex ratios

**Fig.2:**
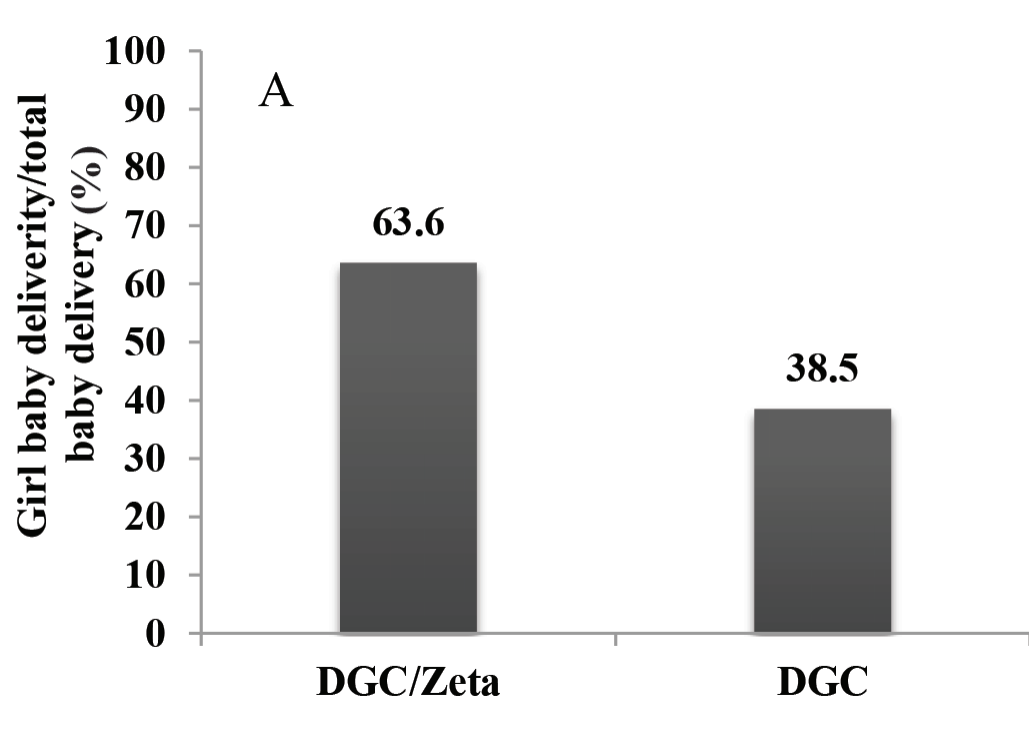
A. Comparison percentage of girl baby delivery to total baby delivery and B. Number of male and female births. ^*^; Indicates statistical significance in sex ratio between two groups. Chi- square carried out for statically analyzing, F; Female, M; Male, and DGC; Density gradient centrifugation.

Figure 2 compares the percentage of girl baby delivery to total baby delivery between DGC and DGC/Zeta groups. As shown, the percentage of girls delivered after DGC/Zeta sperm selection procedure was significantly higher than DGC procedure with a P<0.001. In addition, we observed statistical significance in sex ratio between DGC and DGC/Zeta groups. Sex ratio was significantly lower in the DGC/Zeta group compared to DGC group (P=0.04). Therefore, the number of girl birth was higher in the DGC/Zeta group compared to DGC group.

### X- and Y- bearing sperm ratios

We assessed and compared the ratio of X- and Y- bearing sperm by FISH and RT-qPCR methods in the DGC and DGC/Zeta groups. As depicted in Table 5, the ratios of X- and Y- chromosome bearing sperm population were not significantly different between the two groups. 

**Table 5:** Comparison of X and Y chromosome-bearing sperm populations between washed sperm, DGC and DGC/Zeta groups’ by real-time PCR


Groups	Sample number	Replicate per sample	Ct_SRY_/Ct_Amelo_	P value

Male blood	17	3	0.99 ± 0.01	0.21
washed sperm	17	3	0.99 ± 0.01	0.22
DGC	17	3	0.99 ± 0.01	0.20
DGC/Zeta	17	3	1.00 ± 0.01	0.17


One-way analysis of variance (ANOVA) carried out for statically analyzing. DGC; Density gradient centrifugation , Ct; Cycle threshold and PCR; Polymerase chain reaction.

## Discussion

The association between sperm maturation and chromatin integrity with ICSI outcome is well established in several studies (17-19). Moreover, intensive studies on sperm morphology and chromatin status have revealed that that spermatozoa with apparently normal morphology may have fragmented DNA. Therefore, a simple selection of ICSI sperm based on viability and morphology does not necessarily preclude the chance of oocyte injection with a DNA-damaged or apoptotic sperm (3). This notion has been supported by inverse as- sociation observed between the increased proportion of normal spermatozoa with damaged DNA with embryo quality and also pregnancy outcome after ICSI (20). There are evidence that sperm selected based on their Zeta capacity represent lower degree of DNA damage (6,21). A recent study by Simon et al. (22) showed that selection of negatively-charged sperm through micro-electrophoresis decreased the degree of DNA damage. Therefore, to reduce the chance of selection of morphologically normal spermatozoa with damaged DNA during ICSI, we carried out a double-blind randomized clinical trial to investigate the efficiency of Zeta sperm selection method to distinguish between intact and damaged sperm. 

The results of this study revealed that selection of sperm based on Zeta method increases embryo quality, and chemical and clinical pregnancy rates taking into account all the possible confounding factors which may affect the ICSI outcomes. The confounding factors which had significant influence on the ICSI outcome were male smoking, female age, total numbers of oocyte retrieved and injected, ovarian factors and polycystic ovarian syndrome. These findings are in agreement with the available studies (23,25). Furthermore, the improved ICSI outcomes are consistent with our previous study which suggested that selection of sperm based on sperm functional characteristics reduces the possibility of insemination of DNA damaged sperm during ICSI (4). These results are also in concordance with previous preliminary studies which have implemented SpermSep® CS-10 technique based on sperm surface negative charge (21). To our knowledge, this is the first clinical trial on a large cohort patient group that evaluates the outcome of novel sperm selection based on Zeta potential after ICSI procedure. 

The Zeta potential of human Ybearing sperm has been estimated to be around -16 mV, while the corresponding value for the X-bearing sperm is around -20 mV. The higher negative charge of Xbearing sperm has been attributed to 25% more densely charge sialated proteins residues on their plasma membrane (10). Based on these reports, we compared the sex ratio of children born in DGC and DGC/Zeta groups which was significantly in favor of higher females born in the DGC/Zeta procedure. Subsequently, we analyzed the ratio of Xand Y-bearing sperm using quantitative PCR and FISH analysis. The results revealed no significant difference between the ratios of Xand Y -bearing sperm between the two groups. These results are consistent with previous report of Ainsworth et al. (15) using electrophoretic chamber designed based on sperm Zeta potential to separate sperm with intact DNA. They also reported no significant difference in the ratio of X and Y bearing sperm using quantitative PCR. 

Considering the fact that the study was a doubleblind trial in which the individuals who carried out the ICSI procedure were unaware of sperm selection procedure (DGC or DGC/Zeta), the tentative difference or the skewed sex ratio of children born through Zeta procedure may be attributed to other possible unknown factors. It seems that the difference could be due to higher resistance of Xbearing sperm to stressful conditions. We had previously shown that during Zeta procedure, sperm with negative Zeta potential attached to the positive surface of the tube and the selected sperm underwent a capacitation-like process. This was confirmed by Chlortetracycline (CTC) staining for detection of capacitated sperm and also externalization of phosphatidyl serine (EPS) as an early marker of apoptosis by annexin V staining (26). EPS is attributed to early apoptosis and part of natural process of capacitation and acrosome reaction when two membranes (inner acrosome and sperm lemma) are in the process of fusion. Based on the present data, we proposed that Y -bearing sperm may be less resistant to this process and become immotile during the Zeta procedure. Therefore, following Zeta procedure, we might be selecting Xbearing sperm which may have resisted the Zeta procedure. This proposition is consistent with a previous report which showed that X-bearing sperm are more resistant to stressful conditions like thermal stress (27). 

Literature background regarding changes in sex ratio from fertilization to birth in ART cycles suggest that “In-vitro-culture-induced precocious Xchromosome inactivation together with ICSI-induced decrease in number of trophectoderm cells in female blastocysts may account for preferential female mortality at early post-implantation stages and thereby variations in sex ratios at birth in ART cycles”. Whether selection of normal sperm, by procedures like Zeta or Time-lapse, may help to overcome these *in vitro* induced defects, remains to be explored (28,29). 

## Conclusion

Selection of sperm based on Zeta potential improves ICSI outcome. Furthermore, the sex ratio is tentatively affected in favor of female sex. However, further studies are required to confirm this possibility and the mechanism by which Zeta selection may alters the sex ratio. 
